# Hip arthroscopy versus open surgical dislocation for femoroacetabular impingement: A systematic review and meta-analysis

**DOI:** 10.1097/MD.0000000000005122

**Published:** 2016-10-14

**Authors:** Dagang Zhang, Long Chen, Guanglin Wang

**Affiliations:** a Department of Orthopaedics, The People's Hospital of Guang’an City, Guangan; b Department of Orthopedics, West China Hospital, Sichuan University, Chengdu, China.

**Keywords:** femoroacetabular impingement, hip arthroscopy, meta-analysis, open surgical dislocation

## Abstract

**Background:**

This meta-analysis aims to evaluate the efficacy and safety of hip arthroscopy versus open surgical dislocation for treating femoroacetabular impingement (FAI) through published clinical trials.

**Methods:**

We conducted a comprehensive literature search using PUBMED, EMBASE, and the Cochrane Central Register of Controlled Trials databases for relevant studies on hip arthroscopy and open surgical dislocation as treatment options for FAI.

**Results:**

Compared with open surgical dislocation, hip arthroscopy resulted in significantly higher Nonarthritic Hip Scores (NAHS) at 3- and 12-month follow-ups, a significant improvement in NAHS from preoperation to 3 months postoperation, and a significantly lower reoperation rate. Open surgical dislocation resulted in a significantly improved alpha angle by the Dunn view in patients with cam osteoplasty from preoperation to postoperation, compared with hip arthroscopy. This meta-analysis demonstrated no significant differences in the modified Harris Hip Score, Hip Outcome Score-Activities of Daily Living, or Hip Outcome Score-Sport Specific Subscale at 12 months of follow-up, or in complications (including nerve damage, wound infection, and wound dehiscence).

**Conclusion:**

Hip arthroscopy resulted in higher NAHS and lower reoperation rates, but had less improvement in alpha angle in patients with cam osteoplasty, than open surgical dislocation.

## 1 Introduction

Femoroacetabular impingement (FAI) is one of the most common causes of hip pain in young adults.^[1]^ FAI is associated with abnormal mechanical contact between the rim of the acetabulum and the upper end of the femur, in which femoral-based (cam), acetabular-based (pincer), or combined impingement deformities exist.^[2,3]^ Furthermore, FAI is considered a major etiologic factor in the pathophysiology of secondary hip osteoarthritis.^[2]^

FAI symptoms include hip pain, limitation of movement, and joint damage.^[3]^ Surgical treatment is performed when symptoms are severe or when nonoperative treatment fails.^[4]^ Open surgical dislocation has been previously considered the main surgical treatment option for FAI.^[5]^ This approach allows the surgeon to directly visualize the femoral head and acetabulum, which guarantees the complete correction of the deformity.^[6]^ Hip arthroscopy is a relatively new procedure that is much less invasive than open surgical dislocation because it uses a small incision and an arthoscope (small camera) to repair any damage.^[6]^

Several reviews have compared the efficacy of hip arthroscopy with open surgical dislocation.^[2,4,5]^ Results suggest that the arthroscopic method is associated with less complications and faster rehabilitation rates. More recently, a number of controlled clinical trials on hip arthroscopy versus open surgical dislocation have been reported.^[7–11]^ These quantitative analyses were not included in previous reviews.^[2,4,5]^ Therefore, we conducted this meta-analysis to systematically review clinical trials that investigated the surgical treatment of FAI. This meta-analysis aims to compare the efficacy and safety of hip arthroscopy versus open surgical dislocation for FAI treatment.

## 2 Methods

This meta-analysis was reported according to the guidelines of the Preferred Reporting Items for Systemic Reviews and Meta-analyses. And the ethical approval was not necessary because our meta-analysis was based on data from previously published studies.

### 2.1 Study selection

Two review authors independently searched the Cochrane Central Register of Controlled Trials (CENTRAL, issue 8 of 12, August 2016), PUBMED (1980 to August 2016), and EMBASE (1980 to August 2016) databases using the following keywords: femoroacetabular impingement, surgery, treatment, therapy, complications, adverse effect, randomized controlled trial, and clinical trial. These search terms were combined using the Boolean operator “AND” and “OR” in several combinations. In addition, the following MeSH (Medical Subject Headings) vocabulary headings/subheadings were used: femoracetabular impingement/complications, femoracetabular impingement/surgery, and femoracetabular impingement/therapy.

### 2.2 Inclusion and exclusion criteria

Inclusion criteria were as follows: randomized controlled trials (RCTs) and controlled clinical trials, studies that compared open surgical dislocation with hip arthroscopy, reports on the efficacy or safety of both procedures, and studies that included patients clinically diagnosed with FAI.

Exclusion criteria were as follows: case reports and cohort studies, studies that included FAI patients with previous surgery of the affected hip, and studies that performed open surgery without surgical dislocation such as the modified Smith–Petersen approach.^[12]^ Disagreements on study selection were resolved by discussion and consensus between authors.

### 2.3 Data extraction

Two review authors independently extracted information from eligible studies according to the predefined selection criteria. Relevant data included the name of the first author, publication year, study type, sample size, interventions, length of follow-up, representativeness of cases, selection of controls, definition of controls, comparability of cases and controls, ascertainment of exposure, and the equivalent methods of diagnosis and determination of response rate for cases and controls.

Clinical data that addressed primary and secondary outcome measures were extracted when available. Primary outcome measures were as follows: improvement of the alpha angle by the Dunn view in patients with cam osteoplasty from preoperation to postoperation; Nonarthritic Hip Score (NAHS) at 3 months of follow-up; NAHS improvements from preoperation to 3 months postoperation; and modified Harris Hip Score (mHHS), NAHS, Hip Outcome Score-Activities of Daily Living (HOS-ADL), and Hip Outcome Score-Sport-Specific Subscale (HOS-SSS) at 12 months of follow-up. Secondary outcome measures were as follows: reoperation rate and complication rate (including nerve damage and wound problems). Disagreements on data extraction were resolved by discussion.

### 2.4 Quality assessment

Two review authors independently assessed the quality of each study using the Newcastle-Ottawa Scale (NOS), which consisted of 3 quality parameters: selection (maximum score of 4), comparability (maximum score of 2), and exposure or outcome assessment (maximum score of 3).^[13]^ A score of 9 reflects a study of the highest quality, whereas a score ≤5 reflects a study of relatively low quality. Disagreements on study quality assessment were resolved by discussion.

### 2.5 Risk of bias assessment

Potential publication bias was assessed by Egger's linear regression test.^[14]^ A value of *P* < 0.05 was interpreted as evidence of publication bias.^[14]^

### 2.6 Statistical analysis

Statistical analyses were conducted with Review Manager 5.3 (The Nordic Cochrane Centre, The Cochrane Collaboration) and Stata 12.0. For each study, odds ratio (OR) with 95% confidence interval (CI) was calculated for dichotomous outcomes. Two measures were implemented with associated 95% CIs to assess treatment effects for continuous outcomes: mean difference (MD) for studies with comparable outcome measures and standardized mean difference (SMD) for data with disparate outcome measures.^[15]^

Heterogeneity was assessed by visual inspection of the forest plot, and by χ^2^ and I^2^ tests. An I^2^ value >50% and *P* < 0.1 reflected significant heterogeneity. A fixed-effects model was applied for outcome data with no evidence of significant heterogeneity, whereas a random-effects model was used for outcome data with evidence of significant heterogeneity.^[15]^

Sensitivity analyses that excluded one study at a time were performed to determine whether results were reliable.

## 3 Results

### 3.1 Study characteristics and quality

**Figure 1:**
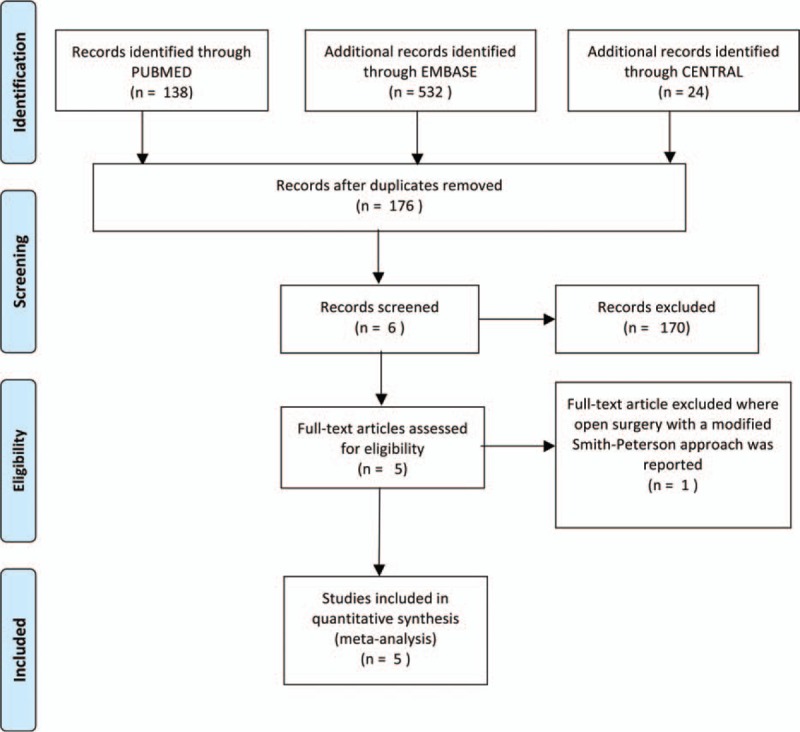
Flow chart of the article screening and selection process is shown. Based on the defined criteria, 5 studies were selected for this meta-analysis.

This search strategy retrieved a total of 694 studies: 24 studies from CENTRAL, 138 studies from PUBMED, and 532 studies from EMBASE. After analyzing titles and abstracts of these references, 6 studies were considered potentially eligible for inclusion.^[7–12]^ One study reported on open surgery with a modified Smith–Petersen approach, and was therefore excluded.^[12]^ The remaining 5 controlled clinical trials met all inclusion criteria for this meta-analysis (Fig. 1). We found no RCTs that compared the efficacy and safety of hip arthroscopy and open surgical dislocation for FAI treatment.

Included studies evaluated a total of 352 hip treatments. Study characteristics are shown in Table 1, including study type, sample size, interventions, and length of follow-up. One study only reported radiographic data from preoperation to postoperation, and did not provide the duration of follow-ups.^[7]^ As shown in Table 2, all studies were considered to be of good or high quality. Based on NOS, one study scored 8 points^[9]^ and one study scored 6 points,^[7]^ whereas the other 3 studies received a score of 7 points.^[8,10,11]^

**Table 1:**
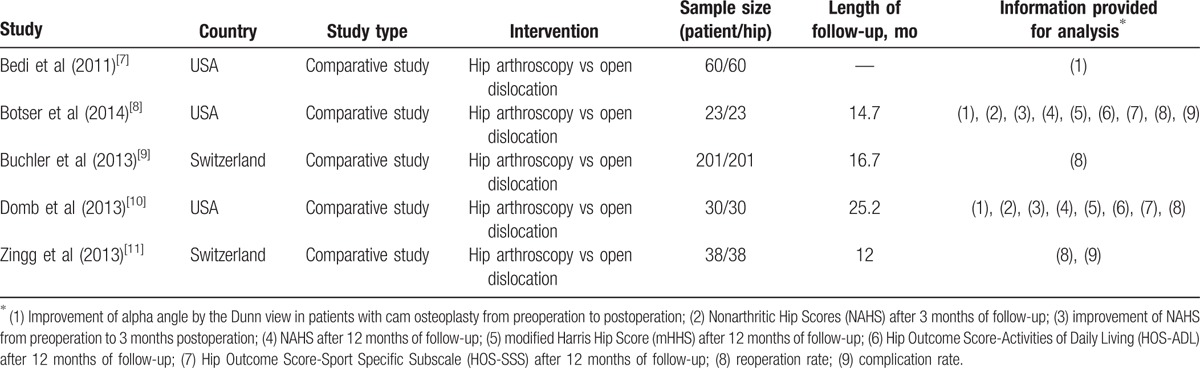
Characteristics of included studies.

**Table 2:**
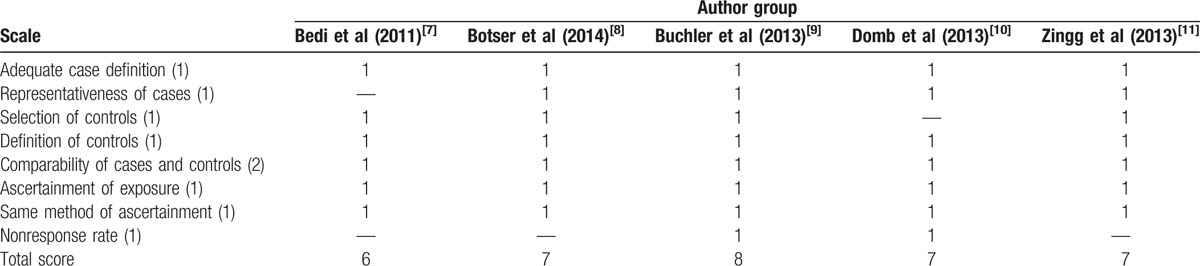
Quality assessment of case-control studies using the Newcastle-Ottawa Scale.

### 3.2 Primary outcome measures

#### 3.2.1 Alpha angle improvement by the Dunn view in patients with cam impingement from preoperation to postoperation

**Figure 2:**
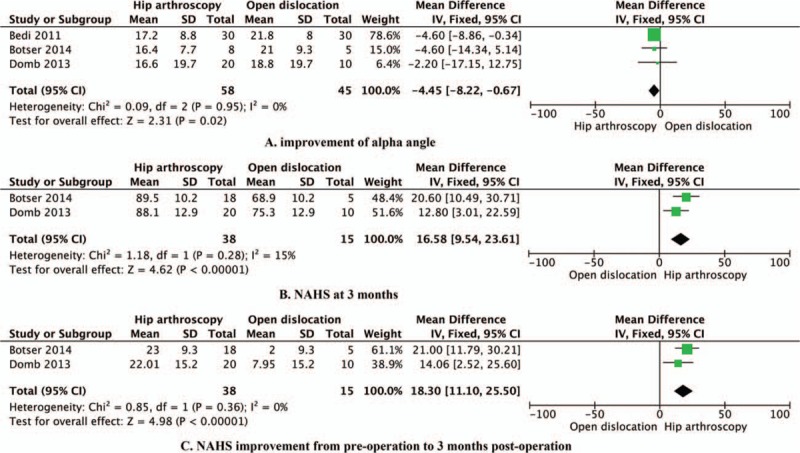
Efficacy of hip arthroscopy versus open surgical dislocation: (A) the alpha angle by the Dunn view in patients with cam osteoplasty from preoperation to postoperation; (B) NAHS at 3 months of follow-up; and (C) improvement of NAHS from preoperation to 3 months postoperation.

Data reporting on alpha angle improvement by the Dunn view in patients with cam impingement from preoperation to postoperation are described in 3 studies^[10]^ that included a total of 103 hips.^[7,8]^ This meta-analysis demonstrated that open surgical dislocation resulted in a significantly improved alpha angle from preoperation to postoperation, compared with hip arthroscopy (−4.45°, 95% CI: −8.22 to −0.67, *P* = 0.02, I^2^ = 0%; Fig. 2A).

#### 3.2.2 NAHS at 3 months

Data reporting on NAHS at 3 months of follow-up are described in 2 studies that included a total of 53 hips.^[8,10]^ This meta-analysis demonstrated that hip arthroscopy resulted in a significantly higher NAHS than open surgical dislocation at 3 months of follow-up (16.58, 95% CI: 9.54–23.61, *P* < 0.00001, I^2^ = 15%; Fig. 2B).

#### 3.2.3 NAHS improvement from preoperation to 3 months postoperation

Data reporting on NAHS improvement from preoperation to 3 months postoperation are reported in 2 studies that included a total of 53 hips.^[8,10]^ This meta-analysis demonstrated that hip arthroscopy resulted in a significantly improved NAHS from preoperation to 3 months postoperation, compared with open surgical dislocation (18.30, 95% CI: 11.10–25.50, *P* < 0.00001, I^2^ = 0%; Fig. 2C).

#### 3.2.4 NAHS, mHHS, HOS-ADL, and HOS-SSS after 12 months

**Figure 3:**
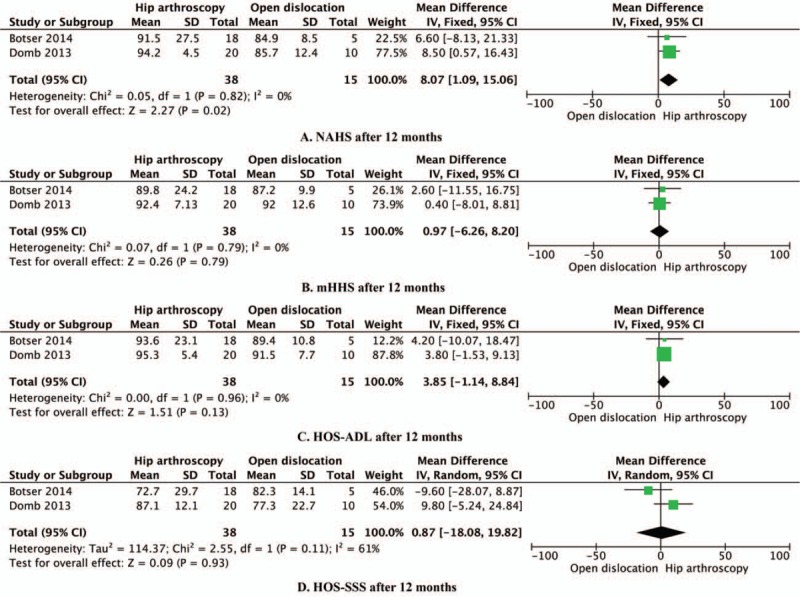
Efficacy of hip arthroscopy versus open surgical dislocation after 12 months of follow-up: (A) NAHS, (B) mHHS, (C) HOS-ADL, and (D) HOS-SSS.

Data reporting on NAHS, mHHS, HOS-ADL, and HOS-SSS at 12 months of follow-up are described in 2 studies that included a total of 53 hips.^[8,10]^ This meta-analysis demonstrated that hip arthroscopy resulted in a significantly higher NAHS than open surgical dislocation at 12 months of follow-up (8.07, 95% CI: 1.09–15.06, *P* = 0.02, I^2^ = 0%; Fig. 3A). No statistical difference was found between hip arthroscopy and open surgical dislocation in mHHS (0.97, 95% CI: −6.26 to 8.20, *P* = 0.79, I^2^ = 0%; Fig. 3B), HOS-ADL (3.85, 95% CI: −1.14 to 8.84, *P* = 0.13, I^2^ = 0%; Fig. 3C), or HOS-SSS (0.87, 95% CI: −18.08 to 19.82, *P* = 0.93, I^2^ = 61%; Fig. 3D) at 12 months of follow-ups.

### 3.3 Secondary outcome measures

#### 3.3.1 Reoperation rate

**Figure 4:**
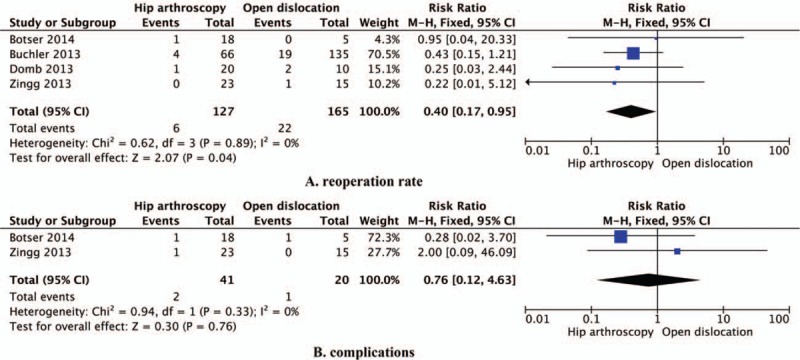
Efficacy and safety of hip arthroscopy versus open surgical dislocation: (A) reoperation rate and (B) complication rate.

Data reporting on reoperation rate are described in 4 studies that included a total of 292 hips.^[8–11]^ This meta-analysis demonstrated that more additional operations were required after open surgical dislocation than after hip arthroscopy (relative risk [RR]: 0.40, 95% CI: 0.17–0.95, *P* = 0.04, I^2^ = 0%; Fig. 4A).

### 3.4 Complications

Data reporting on complications are described in 2 studies that included a total of 61 hips.^[8,11]^ This meta-analysis demonstrated no statistical difference in complications between hip arthroscopy and open surgical dislocation (RR: 0.76, 95% CI: 0.12–4.63, *P* = 0.76, I^2^ = 0%; Fig. 4B).

### 3.5 Sensitivity analysis and publication bias

**Figure 5:**
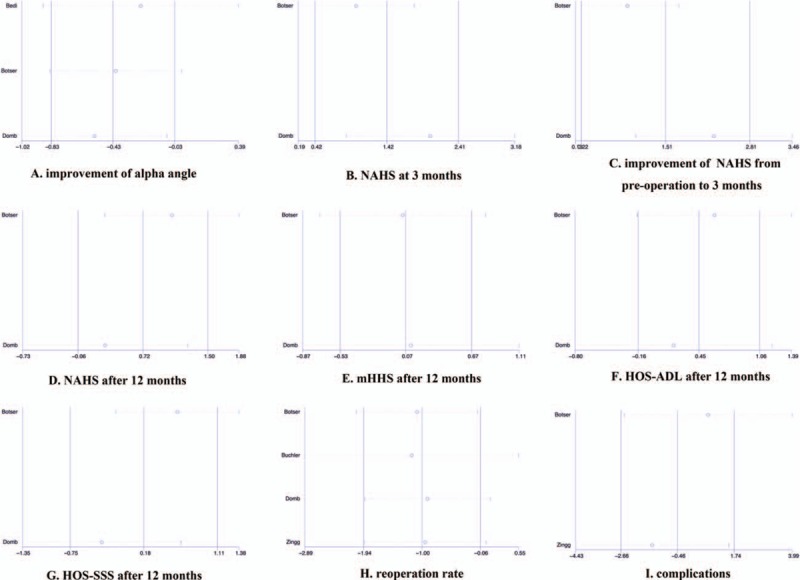
Sensitivity analyses for determining the reliability of results.

Sensitivity analysis was performed to investigate the influence of each individual study on the pooled SMD or OR, excluding one study at a time. Results revealed that no single study significantly affected the pooled SMD or OR (Fig. 5), demonstrating statistically robust results.

Owing to the small number of trials in some analyses, Egger's linear regression test was only performed to assess publication bias in the analyses of alpha angle improvement and reoperation rate. Results in Table 3 revealed that this meta-analysis had no significant publication bias.

**Table 3:**
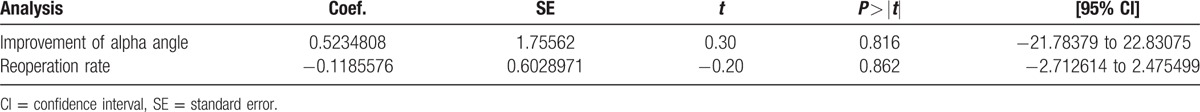
Egger's linear regression test.

## 4 Discussion

This meta-analysis included data from 5 controlled clinical trials involving 352 hips with FAI to evaluate the efficacy and safety of hip arthroscopy versus open surgical dislocation for FAI treatment. Findings presented here revealed that hip arthroscopy resulted in a significantly higher NAHS after 3 and 12 months of follow-up. There was a significant improvement in NAHS from preoperation to 3 months postoperation, and reoperation rate was significantly lower than open surgical dislocation. Conversely, open surgical dislocation results in a significantly improved alpha angle by the Dunn view in patients with cam osteoplasty from preoperation to postoperation, compared with hip arthroscopy. Between these 2 procedures, there were no significant differences in mHHS, HOS-ADL, and HOS-SSS after 12 months of follow-ups, or in complication rate (including nerve damage, wound infection, and wound dehiscence).

Alpha angle is important for evaluating the degree of femoral epiphyseal overgrowth in cam impingement.^[16]^ This meta-analysis found that open surgical dislocation resulted in a significantly improved alpha angle by the Dunn view in patients with cam osteoplasty from preoperation to postoperation, compared with hip arthroscopy. These findings are in contrast to findings reported by Papalia et al,^[17]^ wherein no differences were found in the alpha angle between these 2 methods.

NAHS is frequently used to assess patients with nonarthritic hip pain and recovery of function after hip surgery.^[18]^ Laude et al^[19]^ and Singh and O’Donnell^[20]^ previously reported that postoperative NAHS significantly improved following hip arthroscopy, but this study did not compare the difference of NAHS between hip arthroscopy and open surgical dislocation. Our meta-analysis found that hip arthroscopy resulted in a significantly higher NAHS after 3 and 12 months of follow-up, and a significantly improved NAHS from preoperation to 3 months postoperation, than open surgical dislocation.

Reoperation rate is useful for evaluating the efficacy and safety of any procedure. Harris et al^[21]^ previously reported a significant number of reoperations following surgical dislocation, compared with hip arthroscopy. In accordance with these findings, this meta-analysis found a significantly lower reoperation rate with hip arthroscopy than open surgical dislocation.

Open surgical dislocation was previously considered the primary surgical treatment for FAI.^[5]^ In this meta-analysis, hip arthroscopy was not only associated with better recovery of function, reduction in nonarthritic hip pain, and a lower reoperation rate, but also resulted in less improvement of alpha angle by the Dunn view in patients with cam osteoplasty from preoperation to postoperation, compared with open surgical dislocation. Although hip arthroscopy may require further refinement, it has a potential of becoming a more widely used procedure for FAI treatment.

Our meta-analysis is associated with several limitations. First, only controlled clinical trials were included due to lack of RCTs in this field, which might diminish the significance of the conclusions. Second, studies included in this meta-analysis were identified by electronic searches of the CENTRAL, PUBMED, and EMBASE databases. Although the search strategy was broad and extensive, not all related studies were included; mainly because of publication bias, which may exclude obvious outcome differences between these 2 treatment methods.^[22]^ Third, we included a small number of trials in each analysis, and a lack of treatment-provider blinding may have introduced detection bias. Finally, these included studies did not provide sufficient outcome data (e.g., standard deviation), which lead to the use statistical methods in determining outcome data based on provided information.

This meta-analysis demonstrated that hip arthroscopy resulted in a higher NAHS and a lower reoperation rate, but led to less improvement of alpha angle in patients with cam osteoplasty, than open surgical dislocation.
